# Gamma-retrovirus design for efficacious microRNA-mediated gene knock-down and protein co-expression without compromising titers

**DOI:** 10.1093/narmme/ugaf030

**Published:** 2025-08-12

**Authors:** Romain Vuillefroy de Silly, Bili Seijo, Jimmy Maillard, Patrick Reichenbach, Melita Irving

**Affiliations:** Ludwig Institute for Cancer Research, University of Lausanne and Department of Oncology, Lausanne University Hospital (CHUV), CH-1011 Lausanne, Switzerland; Ludwig Institute for Cancer Research, University of Lausanne and Department of Oncology, Lausanne University Hospital (CHUV), CH-1011 Lausanne, Switzerland; Ludwig Institute for Cancer Research, University of Lausanne and Department of Oncology, Lausanne University Hospital (CHUV), CH-1011 Lausanne, Switzerland; Ludwig Institute for Cancer Research, University of Lausanne and Department of Oncology, Lausanne University Hospital (CHUV), CH-1011 Lausanne, Switzerland; Ludwig Institute for Cancer Research, University of Lausanne and Department of Oncology, Lausanne University Hospital (CHUV), CH-1011 Lausanne, Switzerland

## Abstract

RNA interference (RNAi) is a powerful tool for controlling gene expression. Although stable gene down-regulation in cells can be achieved with viral vectors expressing short hairpin RNAs (shRNAs), incorporating shRNAs into a microRNA (miR) backbone can improve both the efficacy and safety of RNAi by exploiting the endogenous miR-processing machinery. For gamma-retroviral vectors there are two main strategies, LTR (Pol II)- and U6 (Pol III)-driven expression of the miR-based shRNA, but both have limitations in viral titers generated, knock-down efficacy, and/or protein/reporter gene co-expression levels. Here, we sought to evaluate the effectiveness of inserting miR-30a-based shRNA within the psi/packaging-containing intron of the murine stem cell virus (MSCV)-based splice-gag vector (MSGV) for which transcription is LTR-driven. Systematic comparison to previously described designs within the same viral backbone comprising a range of different shRNAs targets revealed maximal viral titer, knock-down and level of protein co-expression upon intronic insertion of miR-based shRNA. In addition, we demonstrated that the strategy can be extended to gene knock-down by wild-type miRs, as demonstrated for miR-30c1 and let-7a. Taken together, we have developed a gamma-retroviral vector design facilitating efficacious miR-mediated gene knock-down and maintaining strong viral titers.

## Graphical Abstract

**Graphical Abstract:**
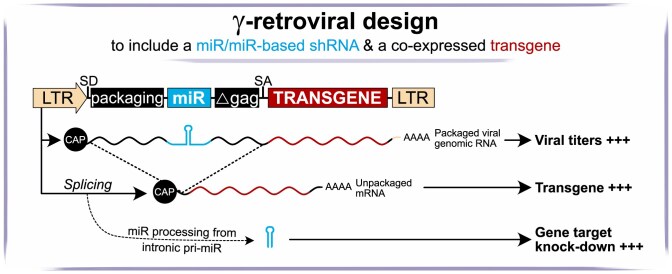

## Introduction

In addition to its critical role in physiological gene regulation, RNA interference (RNAi) has been exploited as a powerful engineering tool [1, 2]. The canonical RNAi pathway involves small interfering RNAs (siRNAs) arising from cleaved double-strand RNAs (dsRNAs) that bind to and degrade sequence-specific messenger RNAs (mRNAs). While synthetic siRNA transfection enables efficient, transient gene knock-downs, stable knock-downs can be achieved using viral vector-based short hairpin RNAs (shRNAs) which generate siRNA *in cellulo* [3]. An important limitation of the latter approach is that shRNA expression is often driven by a powerful Pol III promoter (e.g. U6 and H1) which can lead to cellular toxicities upon saturation of the endogenous microRNA (miR)-processing machinery [4–6].

A strategy to circumvent toxicity associated with Pol III promoter usage is to incorporate the shRNA into a miR [referred to as miR-based shRNAs, shRNAmirs, or amiRs (artificial miRs)], such as the miR-30a backbone [6–13], in order to drive expression from a Pol II promoter. Important advantages of Pol II promoters include that their activity can be modular (i.e. can be regulated/tunable and tissue/cell/stage-specific) and they allow a combination of knock-downs plus the overexpression of reporter genes and/or of therapeutic proteins from a single gene-transcript. A caveat, however, is that the inclusion of miR-based shRNAs within protein-expressing transcripts leads to down-regulation of the protein. This occurs because microprocessor-mediated miR processing promotes either 5′-cap or 3′-poly(A) removal depending on whether the miR is 5′ or 3′ to the protein-coding region, thus potentially disrupting mRNA stability, export to the cytoplasm, and translation. Interestingly, in line with the majority of physiological loci for miRs [14], it was previously shown that efficient protein co-expression can be obtained when miR-based shRNAs are included inside introns [15, 16] given that transcription, splicing, and miR processing are functionally coupled [17].

Both gamma-retroviral and lentiviral vectors for gene-transfer have been commonly and safely used for clinical applications over the past two decades [18, 19] including for chimeric antigen receptor (CAR) T-cell therapy [20]. In order to augment cellular function(s), such as of CAR T cells for better controlling tumors, many pre-clinical studies including our own have demonstrated that T cells can be further co-engineered to over-express immunomodulatory protein(s) [21–23], and/or inhibitory genes can be knocked-down [24, 25]. There remains, however, an important need to develop retroviral vectors enabling optimized combinatorial gene knock-down and protein overexpression, but not at the expense of viral titers. Recently, we developed such a strategy in the context of a lentiviral vector [25], and here we sought to do so in within a gamma-retroviral backbone for use in engineering mouse T cells, tumor-infiltrating lymphocytes, and other cell-types less amenable to infection by lentivirus [23].

While introns present in retroviral vectors are for the most part removed during gamma-retrovirus RNA synthesis in virus-producing cells, the packaging sequence-containing intron is only spliced out upon integration (i.e. post-infection), and in 2014, Park *et al.* demonstrated the feasibility of inserting different miR backbones within this site [26]. Their study, however, was limited by a variability in the results and by the lack of systematic comparison with other strategies. Moreover, there appears to be no follow-up or further use of this approach. Here, in our comprehensive comparative study we show that miR-30a-based shRNAs included in the packaging sequence-containing intron of gamma-retroviruses is in fact the most efficacious strategy available to obtain maximal titer, protein co-expression, and targeted gene knock-down. Furthermore, we demonstrate that this strategy is permissive to gene knock-down by wild-type miRs, as demonstrated for miR-30c1 and let-7a.

## Materials and methods

### Cell culture

The 293T and C1498 cell lines (ATCC) were grown in DMEM medium containing 4.5 g/l glucose, sodium pyruvate, and glutamax (Gibco), supplemented with 10% heat-inactivated fetal bovine serum (PAN Biotech), 10 mM HEPES, 50 U/ml penicillin, and 50 μg/ml streptomycin (Gibco). OT-I CD8^+^ T cells were cultured in DMEM medium containing 1 g/l glucose, sodium pyruvate, and glutamax (Gibco), supplemented with 10% heat-inactivated fetal bovine serum (PAN Biotech), nonessential amino acids, 10 mM HEPES, 50 U/ml penicillin, 50 μg/ml streptomycin, and 50 μM β-mercaptoethanol (Gibco).

### Plasmids

The ecotropic packaging plasmid pCL-Eco was a gift from Inder Verma (Addgene plasmid #12371; http://n2t.net/addgene:12371; RRID: Addgene_12371) [27]. MSGV2W plasmid was derived from the MSGV retroviral vector [28]: NdeI sites prior to BglII sites were removed, the multiple cloning site sequence was removed down to BamHI (which was also destroyed), and a Kozak sequence together with a codon-optimized Thy1.1 and an optimized WPRE version (oPRE) [29] were inserted. A SIN PGK vector was further derived from the MSGV2W plasmid. Briefly, (i) the U3 sequence of the 5’ long terminal repeat (LTR) was replaced with the Rous sarcoma virus (RSV) promoter, (ii) the splicing donor located downstream of the primer binding site was destroyed, (iii) downstream of the psi/packaging sequence the whole truncated/mutated gag sequence was removed [including the splicing acceptor (SA) site], (iv) the human phosphoglycerate kinase (PGK) promoter was added, (v) most of the U3 region from the 3’LTR was deleted, and (vi) the bovine growth hormone polyadenylation (bGHpA) site was added directly after the U5 region of the 3’LTR. In order to obtain CD25-expressing C1498, a UBC SIN vector expressing the mouse CD25 protein was derived from the SIN PGK vector by replacing the PGK promoter by a human ubiquitin C (UBC) promoter and Thy1.1 by CD25.

miR-based shRNA “Pre-gag intron” MSGV2W vector was obtained by inserting the miR-30a backbone at the ApaI site; the miR-based shRNA “Post-gag” MSGV2W vector was obtained by inserting the miR-30a backbone at the BglII site; the miR-based shRNA “Post-gag PGK” MSGV2W vector was obtained by inserting the miR-30a backbone followed by a human PGK promoter at the BglII site; and the miR-based shRNA “SIN U6 PGK” vector was obtained by inserting the human U6 promoter (including the leader sequence [8]) and the miR-30a backbone followed by a poly(6)T transcription terminator at the BglII site (upstream of the PGK promoter). The empty miR-30a backbone (flanked with AgeI and EcoRI sites) was inserted with a PaQCI site in place of the shRNA and loop regions so that to conveniently ligate into the vectors hybridized 67-mer primers that includes the shRNA selected from http://splashrna.mskcc.org/ [30] together with the loop. The most efficient shRNA out of five assessed was selected. The miR-based shRNA against DROSHA was expressed in the same MSGV2W plasmid backbone in which the entire viral sequence from the start of the 5’LTR to the end of the 3’LTR was removed. A U6-driven miR-based shRNA against DROSHA was inserted together with a minimal Simian virus 40 (SV40) origin of replication [31]. The sequences of the hybridized primers to obtain the final miR-based shRNAs were as follows:

miRCTRL-F: AGCGAAGGCAGAAGTATGCAAAGCATTAGTGAAGCCACAGATGTAATGCTTTGCATACTTCTGCCTG

miRCTRL-R: GGCACAGGCAGAAGTATGCAAAGCATTACATCTGTGGCTTCACTAATGCTTTGCATACTTCTGCCTT

miRb2m-F: AGCGCCATCACCTTCTTTATATCTTATAGTGAAGCCACAGATGTATAAGATATAAAGAAGGTGATGT

miRb2m-R: GGCAACATCACCTTCTTTATATCTTATACATCTGTGGCTTCACTATAAGATATAAAGAAGGTGATGG

miRCD25-F: AGCGAACATGTGTAGATGAAAGAGAATAGTGAAGCCACAGATGTATTCTCTTTCATCTACACATGTG

miRCD25-R: GGCACACATGTGTAGATGAAAGAGAATACATCTGTGGCTTCACTATTCTCTTTCATCTACACATGTT

miRCD44-F: AGCGCTGTGTTGACTTTTCAAATATATAGTGAAGCCACAGATGTATATATTTGAAAAGTCAACACAT

miRCD44-R: GGCAATGTGTTGACTTTTCAAATATATACATCTGTGGCTTCACTATATATTTGAAAAGTCAACACAG

miRCD45-F: AGCGCACAGTTTTATGCTTATTTTAATAGTGAAGCCACAGATGTATTAAAATAAGCATAAAACTGTA

miRCD45-R: GGCATACAGTTTTATGCTTATTTTAATACATCTGTGGCTTCACTATTAAAATAAGCATAAAACTGTG

miRCD54-F: AGCGAAGGAGGTGAATGTATAAGTTATAGTGAAGCCACAGATGTATAACTTATACATTCACCTCCTC

mirCD54-R: GGCAGAGGAGGTGAATGTATAAGTTATACATCTGTGGCTTCACTATAACTTATACATTCACCTCCTT

miRDROSHA-F: AGCGCCAGAGATATTTTGGAATTATATAGTGAAGCCACAGATGTATATAATTCCAAAATATCTCTGA

miRDROSHA-R: GGCATCAGAGATATTTTGGAATTATATACATCTGTGGCTTCACTATATAATTCCAAAATATCTCTGG

Vectors to obtain polycistronic Thy1.1-T2A-GFP-miRtarget mRNAs were built in the MSGV2W SIN backbone. The perfect miR-30c (hsa-miR-30c-5p) and let-7a (hsa-let-7a-5p) -5p targets were added straight after the stop codon of a Thy1.1-T2A-GFP sequence whose expression was driven by a PGK promoter. As a control, a Thy1.1-T2A-GFP vector without any miR target was constructed in parallel. miR-expressing vectors were obtained as described above with miR-based shRNAs at the ApaI restriction site except that a mCherry reporter gene was co-expressed instead of Thy1.1. In order to obtain as much physiological processing as possible leading to pri-miR generation, 110–120 bp surrounding the pre-miR sequences (mir-30c-1 and let-7a-1) were included.

### Retrovirus production and C1498 transduction

293T were transfected using the calcium phosphate technique [32] with a 1:1 (2 μg of each for a 9.5 cm^2^ well) retroviral transfer vector to packaging plasmid ratio (when used, 1 μg miRDROSHA was added at the same time for a 9.5 cm^2^ well). Medium was replaced one day later and supernatants were harvested 2 days post-transfection. Retroviral content was titrated (based on CD90.1/Thy1.1 positivity by flow cytometry) by serial dilution of viral supernatants with the C1498 cell line in the presence of 10 μg/ml protamine sulfate (Sigma–Aldrich, P4020). For murine CD8^+^ T cell transduction, viral supernatants from the various designs were obtained by transfecting the constructs in the presence of the miRDROSHA plasmid and were subsequently frozen 2 days post-transfection. CD25-expressing C1498 cell line was obtained upon transduction with a CD25-expressing retrovirus and selection by magnetic separation (CELLection Biotin Binder Kit, ThermoFisher Scientific) via CD25 surface labeling. Polycistronic Thy1.1-GFP-miRtarget-expressing C1498 cell line was obtained upon transduction with MSGV2W retrovirus and selection by magnetic separation (CELLection Biotin Binder Kit, ThermoFisher Scientific) via Thy1.1 surface labeling. At least 7 days post-transduction, purity (>98%) was confirmed by flow cytometry.

### Splenocyte isolation, CD8^+^ selection, and transduction

Splenocytes were extracted from OT‐I mice. CD8^+^ T cells were purified by negative selection using the Biolegend MojoSort mouse CD8^+^ T cell isolation kit (480008; >90% purity) and were primed by culture on plates pre-coated with anti-CD3 antibodies (5 μg/ml; clone 145-2C11, Biolegend) in the presence of anti-CD28 antibodies (1 μg/ml; clone 37.51, Biolegend) at 37°C with 5% CO_2_. After 2 days, cells were transduced in the presence of 10 μg/cm^2^ of Retronectin (Takara) following manufacturer’s instructions and recombinant murine IL-2 was added (200 IU/ml; Peprotech). The next day, cells were transferred to another plate, diluted in the presence of exogenous IL-2, and analyzed by flow cytometry 2 days later to determine viral titers, protein co-expression levels, and knock-down efficacy.

### Flow cytometry

The buffer used for flow cytometry studies was composed of PBS, 0.5% BSA, 0.1% NaN_3_, and the staining procedure included the use of a live/dead fixable cell stain kit (Molecular Probes) to analyze viable cells. For surface marker expression, cells were stained with anti-H-2Kb/H-2Db-PE (114607), CD8α-BV421 (100738), CD25‐APC (102012), CD44‐APC (103011), CD45RB-APC (103319), CD54-APC (116119), and CD90.1/Thy1.1-BV421 (202529) antibodies from BioLegend. Samples were run using a CytoFlex (Beckman Coulter) flow cytometer. Analyses were carried out on singlets using FlowJo software, and marker expression was calculated from the Median Fluorescence Intensity (MFI). Knock-down (KD) efficacy was calculated relative to the MFI obtained with the miRCTRL condition as follows: KD (%) = 100 − (MFI miR of interest × 100/MFI miRCTRL). In order to assess miR efficacy upon one viral copy integration per cell, a titration with miR-expressing viruses was carried out and MFI values were analyzed in conditions for which <25% C1498 cells were transduced.

### Immunoblotting

293T cells were transfected with a plasmid encoding a miR-based irrelevant shRNA (without target: miRCTRL) or targeting DROHSA (miRDROSHA). Cells were lysed in RIPA buffer supplemented with Halt phosphate/protease inhibitors (ThermoFisher Scientific) and were boiled at 97°C for 10 min with Bolt LDS sample buffer and reducing agent (ThermoFisher Scientific). Fifteen micrograms of protein sample was separated by sodium dodecyl sulfate-polyacrylamide gel electrophoresis (SDS–PAGE) and transferred to polyvinylidene fluoride (PVDF) membranes using the iBlot2 system (ThermoFisher Scientific). Antibody staining of DROSHA (CST, 3364) or GAPDH (Progen, 690975) was carried out according to the manufacturer’s instructions. Images were acquired with a western blot imager (Fusion, Vilber Lourmat), and protein levels were quantified using the ImageJ software by analyzing pixel intensity. Total DROSHA was calculated by dividing its signal to the GAPDH. Knock-down percentage was calculated by normalization to the miRCTRL condition.

## Results

### Generation of retroviral vector configurations for expressing miR-based shRNA

**Figure 1.**
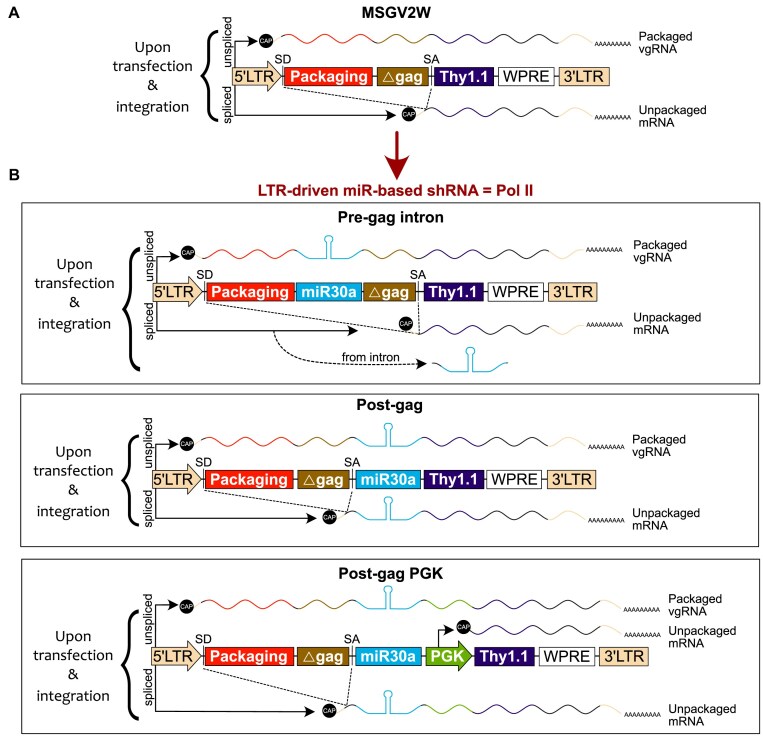
Design of LTR-driven miR-based shRNAs in the MSGV2W gamma-retroviral backbone. (**A**) Schematic of the MSGV2W backbone. (**B**) Schematics of the MSGV2W-derived miR-expressing vectors. Top: the miR-based shRNA is encoded within the intronic region between the psi/packaging and the mutated/truncated gag (Δgag) sequences (pre-gag intron). Middle: the miR-based shRNA is located after the Δgag sequence and SA site (post-gag). Bottom: the miR-based shRNA is inserted following the Δgag sequence and SA site and combined with a downstream PGK promoter (post-gag PGK). LTR: long terminal repeat; SD: splicing donor; SA: splicing acceptor; WPRE: Woodchuck hepatitis virus post-transcriptional regulatory element; PGK: phosphoglycerate kinase promoter; vgRNA: viral genomic RNA.

**Figure 2.**
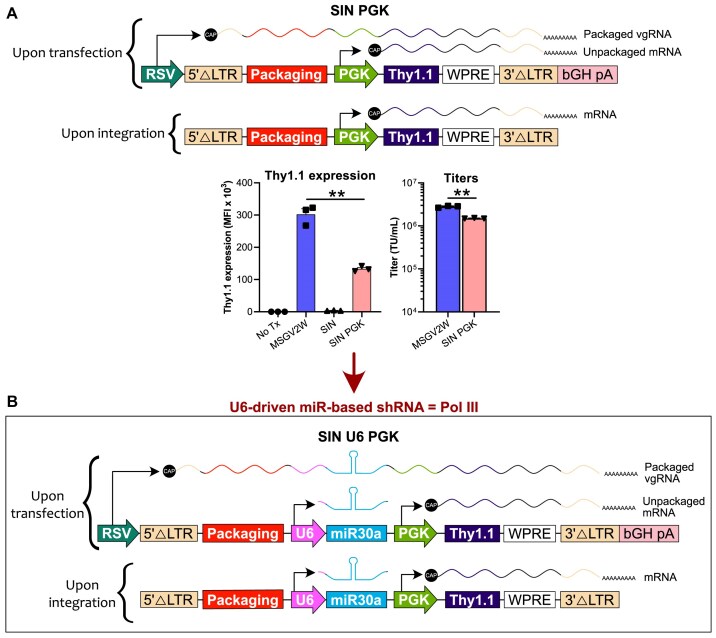
Design of the U6-driven miR-based shRNA in the MSGV2W SIN gamma-retroviral backbone. (**A**) Top: Schematic of the SIN vector backbone generated from MSGV2W and named SIN PGK. Bottom: Bar graphs display mean Thy1.1 expression (left) and mean titers ± SEM (right) from three independent experiments (depicted by symbols; “No Tx”: no transduction) as measured by flow cytometry upon transduction in the C1498 cell line. **: *P* < 0.01 (Student’s paired *t*-test). MFI: median fluorescence intensity; TU: transducing unit. (**B**) Schematic of the SIN-derived miR-expressing vector named SIN U6 PGK. bGH pA: bovine growth hormone polyadenylation site; RSV: Rous sarcoma virus promoter; vgRNA: viral genomic RNA.

We began our study to evaluate the impact of encoding miR-based shRNAs within the packaging sequence-containing intron of gamma-retroviruses on (i) viral titers, (ii) expression levels of a reporter protein, and (iii) knock-down efficiency of the target gene, as compared to currently used configurations. These configurations include encoding the miR-based shRNA in the exonic region, or most commonly within the exonic region along with an exogenous promoter (i.e. the PGK promoter) to elevate reporter protein expression levels (Fig. 1) [12, 33, 34]. In addition, we tested Poll III (i.e. U6 promoter)-driven miR-based shRNA expression along with an exogenous Pol II promoter (i.e. PGK promoter) to drive expression of a reporter protein in a self-inactivating (SIN) retroviral vector (Fig. 2) [8, 34].

Previously, the above-mentioned configurations have been used in a range of vectors including Moloney murine leukemia virus (MMLV), murine stem cell virus (MSCV), and SIN lentiviruses with internal Pol II promoter-driven miR-based shRNA expression. Here, for meaningful comparison, we built all of the vectors with the same gamma-retrovirus backbone, the MSCV-based splice-gag vector (MSGV), which has an intron (enabling high transgene expression) that encompasses the packaging sequence and an extended mutated/truncated gag sequence (Δgag) [28]. While the packaging sequence contains mandatory elements needed for encapsidation/packaging of the viral genomic RNA [35], the Δgag sequence is mostly described to improve it [28, 36]. For all constructs, Thy1.1 was used as a reporter gene, and we included a Woodchuck hepatitis virus post-transcriptional regulatory element (WPRE) to further enhance viral titer and protein expression levels as previously described [29, 37, 38]. We named the modified base construct MSGV2W (Fig. 1A). We then inserted the miR-based shRNAs either between the packaging and the Δgag sequences (“pre-gag intron”; Fig. 1B, top), straight after the Δgag sequence and SA site (“post-gag”; Fig. 1B, middle), or immediately following the Δgag sequence and SA site and combined with a downstream PGK promoter (“post-gag PGK”; Fig. 1B, bottom). Given its wide-use, and the availability of a web-based shRNA efficacy prediction tool (http://splashrna.mskcc.org/) [30], we employed the miR-30a backbone for our study [12, 33].

Similar to previous studies [39–41], we generated high titers for the SIN version of MSGV2W which we named “SIN PGK” (Fig. 2A, top). Briefly, to generate the SIN vector, we replaced the 5’-long terminal repeat (5’-LTR) U3 region with a RSV promoter, removed Δgag, included a human PGK promoter to drive Thy1.1 expression, truncated the 3’-LTR U3 region, and added a bovine growth hormone polyadenylation site (bGH pA) after the 3’-LTR. In accordance with the weaker transcriptional activity of PGK as compared to the LTR, we observed that reporter gene-expression (Thy1.1) levels dropped by ∼50% for SIN PGK versus MSGV2W, but viral titers were quite similar for both (Fig. 2A, bottom) vectors. Subsequently, we inserted a U6 promoter upstream of the PGK promoter [8] in order to express miR-30a-based shRNA (Fig. 2B). We named this vector “SIN U6 PGK.”

### Comparison of viral titers and co-expressed reporter-protein levels

**Figure 3.**
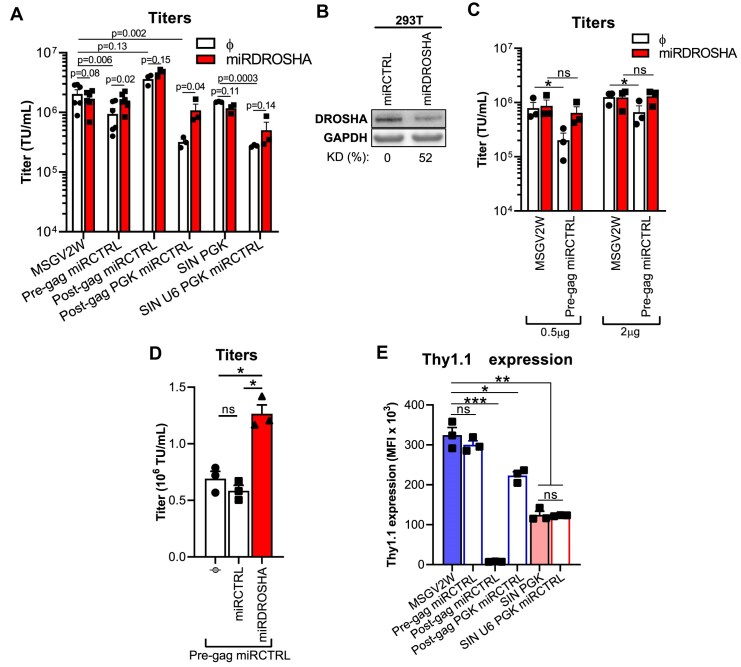
miR-based shRNA included in the intronic region of a gamma-retroviral vector enables high titers of virus and efficient protein co-expression in transduced cells. (**A**) Titers of the different vector designs encoding miR-based shRNA strategies as compared to the parental vectors (MSGV2W and SIN PGK) encoding Thy1.1 only. 293T cells were transfected with the retro-vector plasmids in the presence or absence of a plasmid encoding a miR-based shRNA control (“miRCTRL”) against DROSHA (“miRDROSHA”) to improve viral titers. Bar graph displays mean titers + SEM from at least three independent experiments (depicted by symbols). Exact *P*-values are displayed (Student’s paired *t*-test). (**B**) DROSHA knock-down efficacy in the packaging cell line. 293T were transfected with a plasmid encoding a miR-based shRNA against DROSHA (“miRDROSHA”) or a control (“miRCTRL”). DROSHA and GAPDH levels were analyzed by western blot 48 h post-transfection. KD (%): Percentage of DROSHA knock-down. (**C**) Improvement in viral production by increasing the retro-plasmid dose transfected and by knocking down DROSHA. 293T cells were transfected with the indicated retro-vector plasmids at two doses (0.5 or 2 μg) in the presence or absence of a plasmid encoding a miR-based shRNA against DROSHA (“miRDROSHA”). Retroviral titers were determined in C1498 by flow cytometry. Bar graph displays mean titers + SEM from three independent experiments (depicted by symbols). ns: not significant; *: *P* < 0.05 (Student’s paired *t*-test). (**D**) Restoration of retroviral titers upon miR-based DROSHA knock-down is specific. 293T cells were transfected with the pre-gag miRCTRL vector in the absence (“φ”), or presence of a plasmid encoding a miR-based shRNA control (“miRCTRL”) or a miR-based shRNA against DROHSA (“miRDROSHA”). Retroviral titers were determined in C1498 by flow cytometry. Bar graph displays mean titers + SEM from three independent experiments (depicted by symbols). ns: not significant; *: *P* < 0.05 (repeated measures one-way ANOVA, Tukey’s post hoc test). (**E**) Thy1.1 expression levels upon transduction of C1498 with the different retroviral vectors. Bar graph displays mean Thy1.1 expression + SEM from three independent experiments (depicted by symbols) from the Thy1.1^+^ population. ns: not significant; *: *P* < 0.05, **: *P* < 0.01, ***: *P* < 0.001 (Student’s paired *t*-test).

Having generated our panel of vectors for expressing miR-based shRNA, we next evaluated viral titers and reporter protein expression levels for the different designs (Fig. 3). Upon incorporating a miR-based control shRNA that has no target (“miRCTRL”) we observed significantly lower titers for the “Post-gag PGK” (comprising active LTR and PGK promoters) and “RSV U6 PGK” (including active RSV, U6, and PGK promoters during viral production) vectors (Fig. 3A). This is presumably due, at least in part, to tandem transcriptional interference caused by having more than one active promoter during virus production (i.e. interfering with the level of gamma-retroviral genomic RNA available for packaging) [42]. We have observed similar effects in the context of lentiviral vectors comprising multiple promoters [25]. We also saw slightly lower viral titers for the “Pre-gag” vector. We presumed that this was due to miR processing of the viral genomic RNA by the ribonuclease III enzyme DROSHA impeding proper encapsidation by limiting intact viral genomic RNA availability for virus production [43]. Accordingly, titers could be restored by knocking down DROSHA (Fig. 3A and B) in the packaging cell line (293T) and/or by increasing the quantities of the vector during transfection (Fig. 3C). Restoration was specific to DROSHA knock-down and not to miR processing saturation since equivalent addition of a plasmid encoding a miRCTRL did not improve viral titers (Fig. 3D). With regards to reporter gene expression (Fig. 3E), Thy1.1 levels were markedly lower for the “Post-gag” vector and could be significantly increased by encoding the reporter gene under PGK (“Post-gag PGK” vector). This decrease in the “Post-gag” vector is likely a consequence of DROSHA-mediated processing of the Thy1.1 mRNA that contains the miR, leading to 5’-cap absence, inhibition of nuclear export and protein translation. Remarkably, however, miR inclusion in the packaging/gag intron (“Pre-gag intron” vector) generated similar Thy1.1 expression levels as the MSGV2W vector encoding the reporter gene only. Thus, encoding miR-based shRNA within the packaging sequence-containing intron maintains excellent retroviral titers and enables the highest level of protein co-expression.

### miR-based shRNA knock-down efficacy

**Figure 4.**
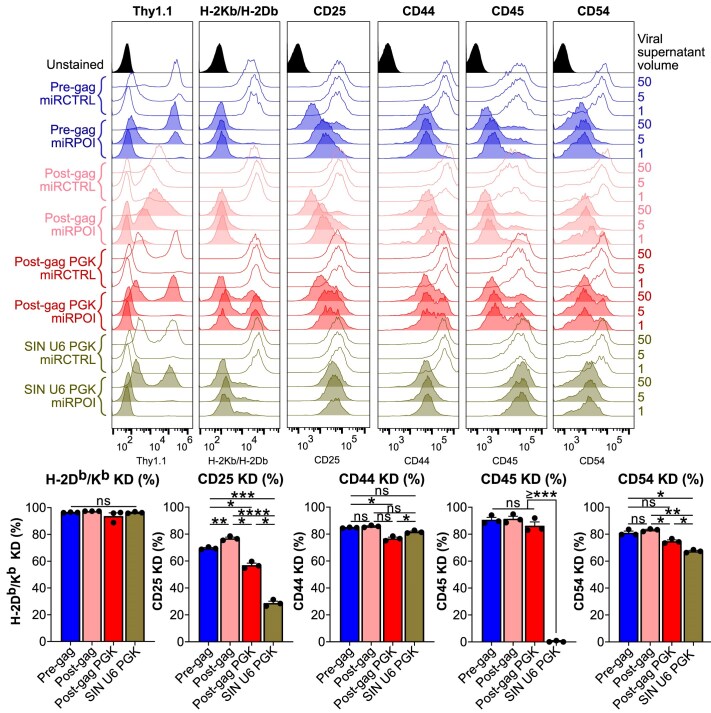
miR-based shRNA included in the intronic region of a gamma-retroviral vector leads to the best knock-down efficiencies, regardless of the target. Flow cytometry histograms of one representative titration experiment are shown upon transduction of C1498. Empty histograms demonstrate the staining upon transduction with miR-based shRNA control (“miRCTRL”) while solid histograms depict transduction with miR-based shRNA against the protein of interest (“miRPOI”). The Thy1.1 histogram (that is matched to the H-2D^b^/H-2K^b^ experiment) reveals Thy1.1 expression together with transduction efficiency for each strategy used. For H-2K^b^/H-2D^b^, CD25, CD44, CD45, and CD54 histograms, only Thy1.1^+^ cells were analyzed. Bar graphs display pooled percent knock-down efficiency + SEM from three independent experiments (depicted by symbols). ns: not significant; *: *P* < 0.05, **: *P* < 0.01, ***: *P* < 0.001, ****: *P* < 0.0001 (repeated measures one-way ANOVA, Tukey’s post hoc test). KD: knock-down.

Next, we sought to evaluate knock-down efficacy for the different vector designs encoding miR-based shRNA. In order to preclude any shRNA-specific bias, we assessed the impact of five shRNAs including for targeting β2-microglobulin, CD25, CD44, CD45, and CD54, in cell line C1498 which we routinely use to determine viral titers. These targets were chosen because they are constitutively expressed by C1498, except for CD25 which we artificially overexpressed, and they can all be easily detected and quantified by flow cytometry. Because differences in virus integration levels may influence miR-based shRNA knock-down efficiency per transduced cell, we set up experimental conditions which should yield a single copy number per cell (i.e. when <25% of target cells are transduced). Interestingly, the most potent configurations were the Pol II-driven miR-based shRNA strategies, in particular the “Pre-gag intron” and “Post-gag” configurations (Fig. 4). Not surprisingly, the Pol III-driven strategy also yielded strong knock-downs with the exception of CD45, which was not impacted at all. Upon careful examination of the sequence of this shRNA we postulate that the presence of two stretches of four thymidines may generate a premature transcriptional stop.

### miR-based shRNA strategies in primary murine CD8^+^ T cells

**Figure 5.**
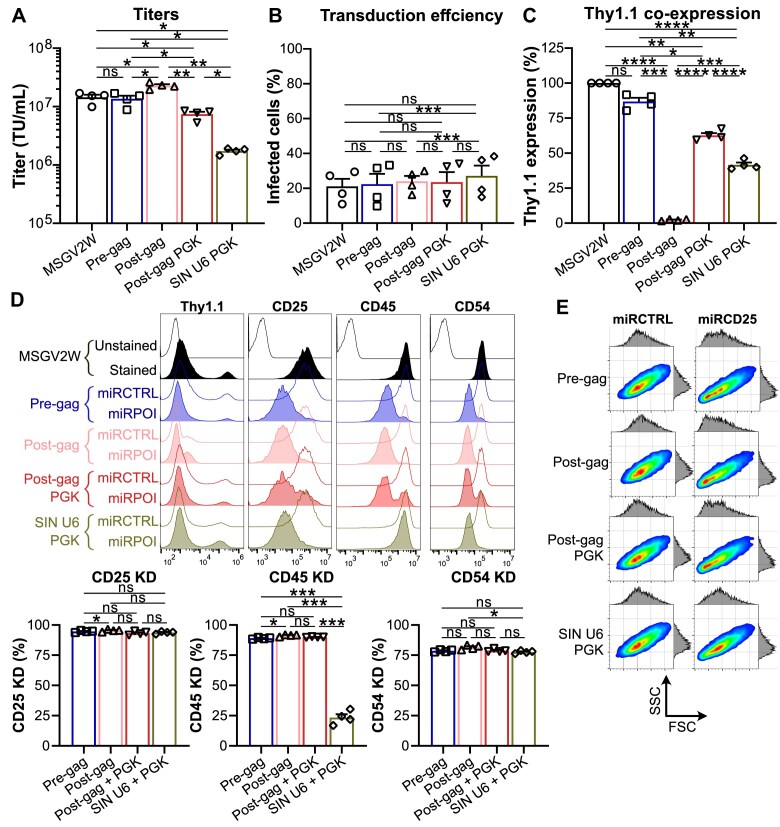
Inclusion of miR-based shRNA within the intronic region of a gamma-retroviral vector (“Pre-gag intron”) is the best design for generating high viral titers along with strong knock-down efficiencies and protein co-expression levels in primary murine CD8^+^ T cells. (**A**) Titers inferred from pre-activated and transduced OT-I CD8^+^ T cells. Viral supernatants were collected upon 293T cells transfection in the presence of a miR-based shRNA against DROSHA to reach maximal viral titers. Upon one freeze and thaw cycle OT-I T cells were transduced and Thy1.1 staining evaluted by flow cytometry was used to infer viral titers. Bar graph displays mean titers + SEM from four biological replicates (symbols) from two independent experiments. ns: not significant; *: *P* < 0.05, **: *P* < 0.01 (repeated measures one-way ANOVA, Tukey’s post hoc test). (**B**) Transduction efficiency in OT-I CD8^+^ T cells. Results show the mean Thy1.1^+^ cells + SEM for four biological replicates (symbols) from two independent experiments as measured by flow cytometry. ns: not significant; ***: *P* < 0.001 (repeated measures one-way ANOVA, Tukey’s post hoc test). (**C**) Thy1.1 co-expression levels upon OT-I CD8^+^ T-cell transduction. Bar graph shows mean Thy1.1 expression (percent MFI) + SEM in the Thy1.1^+^ population for four biological replicates (symbols) from two independent experiments as measured by flow cytometry. ns: not significant; *: *P* < 0.05, **: *P* < 0.01, ***: *P* < 0.001, ****: *P* < 0.0001 (repeated measures one-way ANOVA, Tukey’s post hoc test). (**D**) Knock-down efficiencies for CD25, CD45, and CD54 in OT-I CD8^+^ T cells upon transduction. Flow cytometry histograms show results for one representative biological replicate out of four. Empty histograms represent the staining upon transduction with miR-based shRNA control (“miRCTRL”) while solid histograms depict transduction with miR-based shRNA against the protein of interest (“miRPOI”). The Thy1.1 histogram (that is matched to the CD54 experiment) reveals Thy1.1 expression together with transduction efficiency for each strategy used. For CD25, CD45, and CD54 histograms, cells were gated on Thy1.1^+^. Bar graphs display mean percent knock-down efficiency + SEM for four biological replicates (symbols) from two independent experiments. ns: not significant; *: *P* < 0.05, ***: *P* < 0.001 (repeated measures one-way ANOVA, Tukey’s post hoc test). (**E**) Cell size and granularity of murine OT-I CD8^+^ T cells upon CD25 knock-down. Density plots together with matched FSC (forward scatter)/SSC (side scatter) histograms from one representative biological replicate out of four are displayed.

Given our strong interest in the pre-clinical development of engineered T cells for cancer immunotherapy using syngeneic tumor models, we next compared the efficacy of the differently configured vectors (targeting CD25, CD45, and CD54) in primary murine OT-I CD8^+^ T cells [22, 44]. We observed that titers were much higher (∼10 million transducing units per ml) than the ones calculated using the C1498 cell line, despite one freeze and thaw cycle (Fig. 5A). This is most likely due to the retronectin-based protocol used that increases the proximity between cells and viruses. Importantly, confirming previous results obtained with the C1498 cell line, the “Pre-gag intron” design led to the same titers as the MSGV2W empty vector.

To fairly compare transgene co-expression and protein knock-down we employed a low multiplicity of infection (MOI, Fig. 5B) to analyze the efficacy of each construct at ∼1 viral copy insertion. Thy1.1 levels (Fig. 5C) in the transduced primary murine CD8^+^ T cells were similar to those observed for the C1498 cell line, with the pre-gag intron design yielding the highest Thy1.1 expression. We observed that each design enabled highly effective knock-downs (>75%) for all three proteins analyzed (Fig. 5D). However, confirming previous results in C1498, the U6-driven miR-based shRNA against CD45 was much weaker as compared to the Pol II designs. As expected, given CD8^+^ T cells were cultured in the presence of IL-2 to promote cell expansion, knock-down of CD25, an IL-2 receptor subunit increasing binding affinity for IL-2 leading to enhanced cell enlargement via mTORC1 activity [45, 46], was associated with decreased cell size and granularity (Fig. 5E).

### Leveraging the optimal retroviral design to express miRs

**Figure 6.**
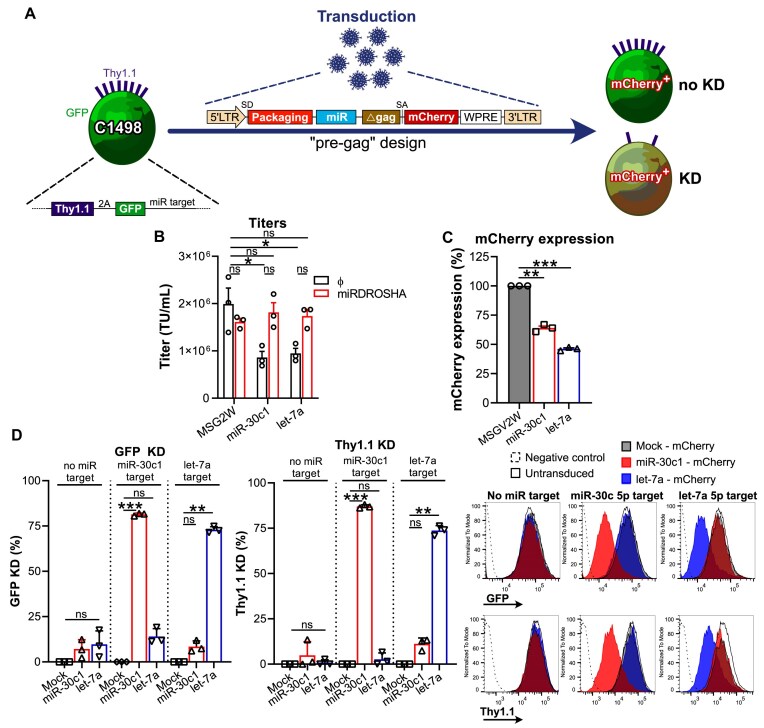
Inclusion of a miR inside the packaging-containing intron of gamma-retroviruses (“Pre-gag intron”) enables effective knock-downs. (**A**) Schematic of strategy to evaluate miR knock-down in the C1498 cell line transduced to express Thy1.1 and GFP as a polycistronic mRNA. Immediately after the GFP stop codon, the complementary miR target (from miR-30c 5p or let-7a 5p) sequence was included. Upon transduction of a miR-expressing vector using the “pre-gag” design that allows mCherry co-expression, knock-down (KD) efficacy was inferred by looking at GFP intensity and Thy1 levels in the mCherry^+^ fraction. (**B**) Titers of miR-containing designs as compared to the parental vector (MSGV2W) encoding mCherry only. 293T were transfected with vectors to produce viruses in the presence or absence of a plasmid encoding a miR-based shRNA against DROSHA. Bar graph displays mean titers + SEM from three independent experiments (depicted by symbols) as measured by flow cytometry upon transduction of C1498. ns: not significant; *: *P* < 0.05 (Student’s paired *t*-test). (**C**) mCherry co-expression levels upon transduction. Bar graph shows mean mCherry expression (percent MFI) + SEM in the mCherry^+^ population from three independent experiments (symbols) as measured by flow cytometry upon transduction of C1498. ns: not significant; **: *P* < 0.01, ***: *P* < 0.001 (Student’s paired *t*-test). (**D**) Knock-down efficacies upon transduction. Bar graphs display mean percent knock-down of GFP or Thy1.1 + SEM in the mCherry^+^ population from three independent experiments (symbols) as measured by flow cytometry. Results are collected according to the genotype of the target cell line transduced (i.e. C1498 that do not have a miR target at the Thy1.1-2A-GFP 3’ tail, those that have a miR-30c 5p target at the 3’ tail, and those that have a let-7a 5p target at the 3’ tail). Histograms show results from one representative experiment upon mCherry^+^ gating (when applicable). Empty histograms with dashed lines represent the signal obtained in wild-type C1498 (“negative control”), empty histograms with solid lines represent the staining in C1498 expressing Thy1.1-2A-GFP untransduced (“untransduced”), gray histograms represent the staining in C1498 expressing Thy1.1-2A-GFP transduced with the empty vector (“mCherry”), red histograms represent the staining in C1498 expressing Thy1.1-2A-GFP transduced with the miR-30c1-expressing vector (“miR-30c1 - mCherry”), and blue histograms represent the staining in C1498 expressing Thy1.1-2A-GFP transduced with the let-7a-expressing vector (“let-7a - mCherry”). ns: not significant; **: *P* < 0.01; ***: *P* < 0.001 (repeated measures one-way ANOVA, Tukey’s post hoc test).

Having demonstrated, for both a cell line and primary murine T cells, the superiority of encoding miR-based shRNA within the packaging-containing intron (“pre-gag”) to current strategies, we next sought to explore knock-down by wild-type miRs in our “pre-gag” vector design. We selected human miR-30c and let-7a as they are both well described and have therapeutic potential [47–52]. With the aim of approaching physiological miR processing we included ∼110 bp flanking regions on both ends of the pre-miRs sequences. In parallel, to assess miR-mediated knock-downs we used a reporter system with ectopic expression of a polycistronic mRNA that encompasses Thy1.1, GFP, and a miR-30c or let-7a target in the C1498 cell line (Fig. 6A). We observed that let-7a was associated with a more pronounced decrease in viral titer and mCherry expression (Fig. 6B and C) than miR-30c1. Nevertheless, inclusion of both miRs enabled effective Thy1.1 and GFP knock-downs (>70%) (Fig. 6D). Hence the “Pre-gag intron” vector design is suitable for knock-down by both miR-based shRNA and wild-type miRs.

## Discussion

Here we sought to design a gamma-retroviral vector for optimally expressing miR-based shRNA along with a protein of interest without compromising virus titers. This is of particular interest to our lab and others for the development of co-engineered CAR- or T cell receptor (TCR)-T cells for cancer immunotherapy [24]. In our study, we systematically compared a largely overlooked strategy in which the miR-based shRNA is encoded in the packaging containing intron [26] to currently used approaches, including in a SIN retroviral vector that we generated incorporating a U6 promoter. For each of the designs we evaluated viral titers, expression levels of the reporter protein Thy1.1, and knock-down efficacy. Because knock-down levels can be dependent on shRNA sequences we targeted five different proteins, β2-microglobulin, CD25, CD44, CD45, and CD54 in cell line C1498, and then three of these (CD25, CD45, and CD54) in primary murine T cells.

Knock-down efficacy is described to be dependent on transcriptional strength of the promoter used [53]. However, despite the strong transcription driven by the U6 promoter, we observed that LTR-driven miR-based shRNAs led to consistently higher target knock-down in our study. In addition to strong transcription by the LTR, one may speculate that Pol II-driven generation of miR-based shRNAs are better processed than the ones arising from Pol III transcription. Indeed, this would be in line with the physiological transcription described for endogenous miRs [54]. Due to the presence of miR-based shRNA during virus production, titers were slightly reduced as a consequence of DROSHA-mediated cleavage of viral genomic RNA. However, this decrease was overcome by knock-down of DROSHA and/or by increasing the amount of plasmid vector transfected in virus-producing cells. Importantly, as previously described [15], levels of a co-expressed protein were not compromised by including a miR-based shRNA inside the intron. We further showed that our “Pre-gag intron” vector strategy can be used to drive knock-down via wild-type miRs. Indeed, inclusion of miR-30c1 or let-7a enabled efficient and specific gene target knock-down, although levels of the co-expressed protein were modestly lowered.

Taken together, we have shown that encoding miR-based shRNA within the packaging intron is the optimal retroviral vector design for reproducibly achieving high viral titers along with strong enforced protein expression levels and knock-down efficacy. Moreover, our strategy is amenable to effective knock-down by wild-type miRs. Work is ongoing to generate “Pre-gag intron” vectors encoding multiple miR-based shRNA or miRs, as well as retroviral particles targeting human T cells and other primary cell-types. We believe that our “Pre-gag intron” vector design will be of strong interest to research groups exploring gene knock-downs in primary cells.

## Data Availability

The data underlying this article are available in Zenodo at https://doi.org/10.5281/zenodo.15913373.
